# Gender Differences in the Clinical and Metabolic Manifestations of Anemia in Chronic Heart Failure

**DOI:** 10.3390/jcm15207961

**Published:** 2026-10-15

**Authors:** Yessen Konysbek, Ayazhan Turar, Vilen B. Molotov-Luchanskiy, Olga A. Ponamareva

**Affiliations:** 1 Department of Internal Diseases, Karaganda Medical University, Karaganda 100028, Kazakhstan; molotov-luchanskiy@qmu.kz (V.B.M.-L.); ponamareva@qmu.kz (O.A.P.); 2 «OTAU MED» LLP, Shymkent 160021, Kazakhstan; ayazhan_turar@mail.ru

**Keywords:** chronic heart failure, anemia, sex differences, iron deficiency, purine metabolism, ejection fraction, ferritin, hypoxanthine, xanthine, hemoglobin

## Abstract

**Background/Objectives:** Anemia and abnormalities in iron-related biomarkers are common in chronic heart failure (CHF), but their relationships with biological sex and left ventricular ejection fraction (LVEF) remain incompletely understood. This study assessed sex-related differences in hematological, iron-related, oxidative stress, and purine metabolism biomarkers across CHF phenotypes. **Methods:** This cross-sectional observational study included 180 patients with CHF, classified according to sex, anemia status based on World Health Organization sex-specific hemoglobin thresholds, and LVEF category: heart failure with preserved ejection fraction (HFpEF), mildly reduced ejection fraction (HFmrEF), or reduced ejection fraction (HFrEF). Hematological parameters, serum iron, ferritin, plasma and erythrocyte purine metabolites, oxidative modification of proteins (OMB), and advanced oxidation protein products (AOPPs) were evaluated. Group comparisons were performed using the Kruskal–Wallis and Mann–Whitney U tests, with Benjamini–Hochberg false discovery rate correction for multiple testing. **Results:** After multiple-testing correction, none of the biomarker comparisons across LVEF categories remained statistically significant when women and men were analyzed separately. Among women with HFpEF, median ferritin was 33.0 μg/L overall and 20.35 μg/L among those with anemia; however, these values could not be used to determine the mechanism of iron deficiency. Among men, anemia was associated with a higher platelet count in HFpEF (287 versus 203 × 10^9^/L; *q* < 0.009) and lower serum iron concentrations in HFpEF (8.8 versus 18.1 μmol/L; *q* = 0.029) and HFrEF (5.5 versus 12.0 μmol/L; *q* = 0.036). In direct comparisons between sexes, only the higher hemoglobin concentration in men with HFmrEF remained statistically significant after correction (148 versus 121 g/L; *q* < 0.036). Nominal differences in plasma purines, hypoxanthine, and xanthine between anemic men and women with HFrEF did not remain statistically significant after correction (all *q* = 0.147). **Conclusions:** The findings demonstrate exploratory associations between sex, anemia status, LVEF category, and selected laboratory biomarkers but do not establish sex-specific mechanisms of anemia or purine metabolism alterations in CHF. Because transferrin saturation, transferrin-related indices, soluble transferrin receptor, hepcidin, and inflammatory biomarkers were not measured, the observed iron-related patterns cannot be classified as absolute iron deficiency or inflammation-associated functional iron restriction. Larger studies incorporating formal interaction testing, multivariable adjustment, and comprehensive assessment of iron status are required.

## 1. Introduction

### 1.1. Background and Clinical Context

Chronic heart failure (CHF) remains a major cause of morbidity, recurrent hospitalization, impaired quality of life, and mortality worldwide [1]. Anemia is a common comorbidity in CHF and is associated with reduced exercise capacity, a greater symptom burden, and poorer clinical outcomes [2]. Anemia and iron deficiency are related but distinct conditions: iron deficiency may occur without anemia, whereas anemia in CHF may result from several overlapping mechanisms, including impaired erythropoietin production or responsiveness, renal dysfunction, inflammation-associated iron restriction, depletion of iron stores, hemodilution, nutritional deficiencies, occult blood loss, bone marrow dysfunction, and treatment-related factors [2].

Inflammation, oxidative stress, and altered cellular energy metabolism may contribute to the development and clinical consequences of anemia in CHF. However, their relative contributions are likely to vary according to heart failure etiology, disease severity, renal function, comorbidity burden, age, sex, and treatment. Disturbances in purine metabolism may reflect altered energy homeostasis and oxidative or hypoxic stress, but their mechanistic role in CHF-associated anemia remains insufficiently established [3]. Accordingly, associations between hematological, iron-related, oxidative stress, and purine metabolism biomarkers should not, in the absence of direct mechanistic measurements, be interpreted as evidence of a specific causal pathway.

It is also important to distinguish between sex and gender. Sex refers to biological characteristics, including chromosomal, hormonal, anatomical, and physiological factors, whereas gender refers to socially constructed roles, behaviors, identities, and exposures that may influence health and healthcare utilization [4]. Because the present study compares biologically defined groups of men and women and examines laboratory and physiological variables, the term “sex differences” is used consistently throughout this manuscript.

### 1.2. Sex-Related Heterogeneity of CHF Phenotypes

The distribution and clinical characteristics of CHF phenotypes differ between women and men. Women are more frequently represented among patients with heart failure with preserved ejection fraction (HFpEF), whereas men more commonly develop heart failure with reduced ejection fraction (HFrEF) [5,6]. Women with CHF are generally older and more frequently have hypertension, obesity, diabetes, and other metabolic comorbidities, while men more often have an ischemic etiology and a history of coronary revascularization. Differences in age, etiology, renal function, comorbidity burden, and treatment may influence hematological and metabolic biomarkers and must therefore be considered when interpreting apparent sex-related differences. Sex-related variation has also been reported in established heart failure biomarkers. Circulating concentrations of natriuretic peptides and galectin-3 tend to be higher in women, whereas cardiac troponins and soluble ST2 are often higher in men [7]. Nevertheless, these differences cannot be attributed to biological sex alone because they may also reflect differences in myocardial mass, body composition, kidney function, heart failure phenotype, disease etiology, and medical therapy.

Inflammation is relevant to CHF pathophysiology in both sexes, and the available evidence does not support assigning an inflammation-driven phenotype exclusively to women. In the PROMIS-HFpEF cohort, Shah et al. identified sex-related proteomic correlates of coronary microvascular dysfunction: inflammation-related chemokine and cytokine pathways were more prominent among men, whereas pathways related to ventricular remodeling and fibrosis were more prominent among women [8]. Other evidence suggests that increased inflammatory activity may have a stronger adverse prognostic association in men with heart failure [9]. Experimental and clinical studies also indicate that sex hormones may modify immune responses, including innate immune activation, antibody and autoantibody production, inflammation, and fibrosis. However, these relationships are complex and may vary according to age, menopausal status, heart failure etiology, and comorbid disease [10]. Taken together, these findings indicate that CHF is biologically and clinically heterogeneous with respect to sex. However, observed differences between women and men should be interpreted as associations unless they are confirmed by formal interaction testing and analyses adjusted for age, renal function, body mass index, diabetes, hypertension, ischemic etiology, functional class, and treatment.

### 1.3. Sex-Related Features of Anemia and Iron-Related Biomarkers in CHF

The World Health Organization defines anemia using sex-specific hemoglobin thresholds: <130 g/L in adult men and <120 g/L in non-pregnant adult women [11]. These thresholds reflect biological differences in hemoglobin distributions but do not establish the underlying cause of anemia. In patients with CHF, anemia is a heterogeneous condition that may be associated with iron deficiency, renal dysfunction, inflammation, nutritional deficiencies, occult blood loss, hemodilution, bone marrow dysfunction, or treatment-related factors. Iron deficiency is clinically relevant in heart failure regardless of whether anemia is present. It has traditionally been defined as a serum ferritin concentration < 100 μg/L or a ferritin concentration of 100–299 μg/L combined with transferrin saturation (TSAT) < 20% [12,13]. However, ferritin is an acute-phase reactant and may be influenced by inflammation, liver disease, renal dysfunction, and other comorbidities. Contemporary evidence increasingly emphasizes TSAT as an indicator of circulating iron availability and as a potential predictor of response to intravenous iron therapy [12]. Serum iron alone is biologically variable and, when considered together with ferritin but without TSAT, transferrin or total iron-binding capacity, soluble transferrin receptor, hepcidin, and inflammatory markers, cannot reliably distinguish absolute depletion of iron stores from inflammation-associated iron restriction.

Previous studies have reported sex-related differences in hemoglobin concentrations and iron-related biomarkers in patients with CHF. Nevertheless, these differences may be influenced by age, menopausal status, renal function, comorbidities, bleeding risk, nutritional status, heart failure etiology, and treatment. Therefore, the available evidence does not justify assigning a predominantly functional iron-deficiency mechanism to women and an absolute iron-deficiency mechanism to men solely on the basis of serum iron and ferritin concentrations. The therapeutic implications of iron deficiency across the LVEF spectrum also remain under investigation. The 2023 ESC focused update and recent trials, including HEART-FID, FAIR-HFpEF, and FAIR-HF2, have expanded the evidence base for intravenous iron therapy but have also demonstrated variation in treatment effects according to patient characteristics, heart failure phenotype, iron-deficiency criteria, and the selected outcomes [13,14,15,16]. These studies do not currently provide a basis for sex-specific diagnostic thresholds or treatment algorithms. Accordingly, the present study focuses on observed sex-related differences in hemoglobin, serum iron, and ferritin rather than inferring functional or absolute mechanisms of iron deficiency.

### 1.4. Purine Metabolism as a Potential Marker of Metabolic Stress

Purine metabolism is closely linked to cellular energy homeostasis. During adenosine triphosphate degradation, hypoxanthine and xanthine are generated and may subsequently be converted to uric acid. Alterations in these metabolites have been reported in cardiovascular disease and may be associated with impaired energy metabolism, oxidative stress, tissue oxygen imbalance, and xanthine oxidase activity [3,17]. However, circulating purine metabolite concentrations are also influenced by renal function, diet, body composition, medication use, and other comorbidities. They should therefore not be regarded as direct or specific measures of myocardial hypoxia or metabolic decompensation. Multi-omics studies in dilated cardiomyopathy have identified associations between myocardial remodeling and pathways involved in purine biosynthesis and catabolism, including pathways related to IMPDH1, ENTPD2, AK7, AK2, and CANT1 [3]. Depletion and altered turnover of the adenine nucleotide pool have also been linked to impaired cardiac energetics and contractile function [17]. Nevertheless, these findings do not establish that circulating purine metabolites directly reflect the severity or progression of CHF. Because anemia reduces the oxygen-carrying capacity of the blood and CHF may impair tissue perfusion, an association between anemia, heart failure phenotype, and purine metabolism biomarkers is biologically plausible. Whether this association differs by sex or LVEF remains uncertain. In the present cross-sectional study, purine metabolites were therefore evaluated as exploratory biomarkers of metabolic stress rather than as direct indicators of hypoxia, disease progression, or a specific causal mechanism.

### 1.5. Knowledge Gap and Study Aim

Although sex-related differences in CHF phenotypes, comorbidities, hematological characteristics, and biomarker profiles have been described, few studies have simultaneously examined the associations of sex, anemia status, and LVEF category with iron-related and purine metabolism biomarkers within a single cohort. Furthermore, differences observed in separate male and female subgroup analyses do not themselves establish sex-specific effects; such effects require formal interaction testing and adjustment for relevant confounding variables. The primary aim of this study was to examine the associations of sex, anemia status, and LVEF category with hematological, iron-related, purine metabolism, and oxidative stress biomarkers in patients with CHF. A secondary exploratory aim was to assess whether the associations between anemia status and the investigated biomarker profiles varied according to sex and LVEF category. Given its cross-sectional design, this study was intended to identify associations and generate hypotheses rather than establish causal mechanisms, temporal progression, or sex-specific diagnostic and therapeutic recommendations.

## 2. Materials and Methods

### 2.1. Study Design and Setting

This analytical cross-sectional observational study was conducted at NCJSC “Karaganda Medical University”, Karaganda, Kazakhstan, within the research protocol entitled “Clinical and Pathogenetic Characteristics of Anemia of Chronic Disease in Patients with Heart Failure”. Consecutive adult patients with chronic heart failure (CHF) were assessed during the study period. This study is reported in accordance with the Strengthening the Reporting of Observational Studies in Epidemiology (STROBE) recommendations. This study was designed to examine the associations of sex, anemia status, and left ventricular ejection fraction (LVEF) category with hematological, iron-related, purine metabolism, and oxidative stress variables. Because all clinical and laboratory variables were assessed at a single time point, this study was intended to identify associations and generate hypotheses rather than establish causal mechanisms or temporal changes.

### 2.2. Study Population

Adult patients were eligible for inclusion if they met the following criteria: age ≥ 18 years; a clinically and instrumentally confirmed diagnosis of CHF according to the 2021 European Society of Cardiology guidelines; available transthoracic echocardiographic assessment, including LVEF; and sufficient laboratory data for the planned hematological, iron-related, and purine metabolism analyses. Patients were enrolled irrespective of hemoglobin concentration. Anemia status was determined after enrollment and was used as an analytical classification variable. The exclusion criteria were acute blood loss, hemolytic anemia, active malignancy, and an acute infectious process at the time of assessment. These conditions were excluded because they could independently affect hematological, iron-related, and metabolic variables. A total of 179 patients with CHF were enrolled and classified according to sex, anemia status, and LVEF category, resulting in 12 analytical subgroups. The number of observations varied slightly between biomarkers because some laboratory measurements were unavailable for individual participants. Therefore, the denominator is reported separately for each variable and comparison, and all analyses were conducted using available cases. No missing values were imputed. The sample size was determined pragmatically by the number of eligible patients enrolled during the study period. No a priori sample-size or statistical-power calculation was performed. Consequently, several subgroups included only 8–18 participants, and all subgroup findings were considered exploratory. The requirement for sufficient laboratory data may also have introduced selection bias by excluding patients without the biomarker measurements required for the present analysis.

### 2.3. Definition of Anemia and Assessment of Iron-Related Biomarkers

Anemia was defined according to the sex-specific World Health Organization thresholds as a hemoglobin concentration < 130 g/L in men and <120 g/L in non-pregnant women. These thresholds were used only to classify anemia status and were not used to determine the underlying mechanism of anemia. Serum iron and ferritin were analyzed as iron-related biomarkers. Transferrin saturation, transferrin, total iron-binding capacity, soluble transferrin receptor, and hepcidin were not available. Therefore, iron deficiency was not classified as functional or absolute, and serum iron and ferritin were not interpreted as definitive evidence of inflammation-mediated iron sequestration or absolute depletion of iron stores.

### 2.4. Diagnosis of CHF and Definition of LVEF Categories

CHF was diagnosed on the basis of symptoms and/or signs consistent with heart failure together with instrumental evidence of a structural or functional cardiac abnormality, in accordance with the 2021 ESC guidelines. Clinical assessment and transthoracic echocardiography were used to establish the diagnosis. Patients were classified into three LVEF categories: heart failure with preserved ejection fraction (HFpEF; LVEF ≥ 50%), heart failure with mildly reduced ejection fraction (HFmrEF; LVEF 41–49%), and heart failure with reduced ejection fraction (HFrEF; LVEF ≤ 40%). HFpEF was diagnosed on the basis of an LVEF ≥ 50% together with symptoms and/or signs of heart failure and available objective evidence of cardiac structural or functional abnormalities. Thus, LVEF alone was not considered sufficient to establish the diagnosis of HFpEF. LVEF was determined by transthoracic echocardiography using the biplane Simpson method.

### 2.5. Clinical and Laboratory Data Collection

Demographic and clinical data included age, sex, body mass index, New York Heart Association functional class, diabetes mellitus, arterial hypertension, renal function, and other documented comorbidities. Information regarding ischemic or non-ischemic heart failure etiology, previous coronary revascularization, chronic kidney disease, autoimmune disease, previous malignancy or chemotherapy, clinically documented bleeding, menopausal status, and anemia-related therapy was not consistently available for all participants. These variables could therefore not be comprehensively included in the subgroup analyses and were considered potential sources of residual confounding.

Hematological variables included hemoglobin, hematocrit, red blood cell count, white blood cell count, and platelet count. These measurements were performed using a routine automated hematology analyzer in the clinical laboratory. Iron-related variables included serum iron and ferritin. Renal function was assessed using serum creatinine and estimated glomerular filtration rate. Creatinine and estimated glomerular filtration rate were classified as renal variables and not as inflammatory biomarkers. C-reactive protein was not included in the reported analyses; consequently, this manuscript does not use CRP to support an inflammation-related mechanism.

Purine metabolism variables included purines, hypoxanthine, adenine, xanthine, and uric acid measured separately in plasma and erythrocytes. Plasma and erythrocyte measurements were treated as distinct variables in all analyses and are reported separately in the revised tables. Oxidative stress variables included protein carbonyl content, referred to as oxidative modification of proteins (OMB), and advanced oxidation protein products (AOPPs). The measurements were performed according to the standardized laboratory procedures used at the study center. Detailed information regarding the analytical platform, sample preparation, calibration, assay variability, and quality-control procedures was not retained in the available analytical dataset. This limitation was acknowledged when interpreting the reproducibility of the biomarker findings.

### 2.6. Ethical Approval

This study was conducted in accordance with the Declaration of Helsinki. The research protocol entitled “Clinical and Pathogenetic Characteristics of Anemia of Chronic Disease in Patients with Heart Failure” was reviewed and approved by the Bioethics Committee of NCJSC “Karaganda Medical University” (Meeting No. 11, Protocol No. 11, approval No. 58; 22 April 2022). Written informed consent was obtained from all participants before enrollment.

### 2.7. Statistical Analysis

Categorical variables were summarized as frequencies and percentages. Continuous variables were presented as medians because most laboratory variables showed non-normal distributions and several analytical subgroups were small. Unadjusted comparisons across the three LVEF categories were performed using the Kruskal–Wallis test. When the overall test indicated a statistically significant difference, pairwise comparisons were conducted using the Mann–Whitney U test. The Mann–Whitney U test was also used to compare patients with and without anemia within each sex and LVEF category and to compare men and women within each LVEF category. Because no single confirmatory biomarker endpoint had been prespecified, all biomarker analyses were considered exploratory. To address the large number of comparisons, *p*-values were adjusted using the Benjamini–Hochberg false-discovery-rate procedure within the hematological and iron-related, purine metabolism, and oxidative stress biomarker families. Both unadjusted *p*-values and adjusted *q*-values were reported. A two-sided *q*-value < 0.05 was considered statistically significant. The results that were significant only before correction for multiple testing were described as nominal associations and were not used to support definitive mechanistic conclusions.

Formal sex × anemia, sex × LVEF, and sex × anemia × LVEF interaction analyses and multivariable adjustment could not be performed using the available aggregated analytical dataset. Therefore, significance in one subgroup and non-significance in another were not interpreted as evidence of a statistically confirmed sex-specific effect. The results were considered descriptive and hypothesis-generating, and potential confounding by age, renal function, body mass index, comorbidities, heart failure etiology, functional class, and treatment was explicitly acknowledged.

## 3. Results

### 3.1. Study Population and Data Availability

A total of 179 patients with chronic heart failure were included in the analysis. The study population comprised 74 women (41.3%) and 105 men (58.7%). According to LVEF category, 53 patients (29.6%) had HFpEF, 64 (35.8%) had HFmrEF, and 62 (34.6%) had HFrEF.

Anemia was present in 72 patients (40.2%). Among women, 39 of 74 patients (52.7%) had anemia, compared with 33 of 105 men (31.4%). In women, anemia was identified in 18 of 26 patients with HFpEF (69.2%), 13 of 26 patients with HFmrEF (50.0%), and 8 of 22 patients with HFrEF (36.4%). In men, anemia was identified in 11 of 27 patients with HFpEF (40.7%), 9 of 38 patients with HFmrEF (23.7%), and 13 of 40 patients with HFrEF (32.5%). These proportions are descriptive and should not be interpreted as evidence of a statistically confirmed sex-specific association because formal interaction testing was not available (Table 1).

Data availability varied slightly across the investigated biomarkers. Hemoglobin and platelet measurements were available for all 179 patients. Serum iron and ferritin measurements were available for 178 patients, whereas purine metabolism and oxidative stress measurements were available for 177 patients. White blood cell and red blood cell measurements in the available dataset were limited to the female subgroup. Therefore, the denominator is reported separately for each variable and comparison, and analyses were conducted using available cases without imputation of missing values.

### 3.2. Laboratory Biomarkers Across LVEF Categories

Laboratory biomarkers across the three LVEF categories are presented separately for women and men in Table 2. Because several measurements were unavailable for individual participants, analyses were performed using available cases, and the corresponding sample size is reported for each subgroup. The reported *p*-values were obtained from the Kruskal–Wallis test, whereas *q*-values were calculated using the Benjamini–Hochberg false discovery rate correction across the complete set of biomarkers evaluated separately in women and men.

Among women, platelet count differed nominally across the LVEF categories, with median values of 276.5 × 10^9^/L in HFpEF, 217.0 × 10^9^/L in HFmrEF, and 202.5 × 10^9^/L in HFrEF (unadjusted *p* = 0.006). However, this association did not remain statistically significant after correction for multiple comparisons (*q* = 0.084). Unadjusted pairwise comparisons suggested lower platelet counts in women with HFmrEF and HFrEF than in those with HFpEF (*p* = 0.036 and *p* = 0.012, respectively); these findings should be interpreted as exploratory.

Hemoglobin concentration also showed a nominal difference among women, increasing from a median of 111 g/L in HFpEF to 121 g/L in HFmrEF and 132 g/L in HFrEF (unadjusted *p* = 0.035; *q* = 0.168). Similarly, median ferritin concentrations were 33.0 μg/L, 90.5 μg/L, and 127.15 μg/L in the HFpEF, HFmrEF, and HFrEF groups, respectively (unadjusted *p* = 0.036; *q* = 0.168). Neither association remained statistically significant following false discovery rate correction. Serum iron did not differ significantly across the female LVEF groups (unadjusted *p* = 0.071; *q* = 0.248). No statistically significant differences were identified in leukocyte or erythrocyte counts, plasma purine metabolites, or oxidative stress biomarkers among women.

Among men, hemoglobin concentration differed nominally across the LVEF categories, with median values of 132 g/L in HFpEF, 148 g/L in HFmrEF, and 141 g/L in HFrEF (unadjusted *p* = 0.021; *q* = 0.132). An unadjusted pairwise comparison indicated a difference between the HFpEF and HFmrEF groups (*p* = 0.018). Serum iron concentrations were 13.3 μmol/L in HFpEF, 15.3 μmol/L in HFmrEF, and 9.5 μmol/L in HFrEF (unadjusted *p* = 0.022; *q* = 0.132), with an unadjusted pairwise difference between HFmrEF and HFrEF (*p* = 0.018). These associations did not remain statistically significant after correction for multiple testing. Platelet count, ferritin, plasma purine metabolites, and oxidative stress biomarkers showed no significant differences across the male LVEF categories.

Thus, several hematological and iron-related biomarkers showed nominal differences across LVEF categories, but none met the prespecified false-discovery-rate threshold after multiplicity correction. These results should therefore be interpreted as exploratory descriptive associations rather than evidence of LVEF-dependent biological or mechanistic differences.

### 3.3. Laboratory Biomarkers According to Anemia Status Within Sex and LVEF Categories

Comparisons of laboratory biomarkers between patients with and without anemia within each sex and LVEF category are presented in Table 3. The analyses were based on available cases. The Benjamini–Hochberg procedure was applied separately in women and men across the complete set of 36 comparisons. Because exact *p*-values below 0.001 were unavailable, the corresponding adjusted values are reported as upper bounds. These subgroup analyses were exploratory and were not considered evidence of sex-specific effects in the absence of formal interaction testing.

Among women, median hemoglobin concentration was lower in patients classified as anemic than in those without anemia in all three LVEF categories: 102 versus 131 g/L in HFpEF, 103 versus 136 g/L in HFmrEF, and 103.5 versus 141.5 g/L in HFrEF (all unadjusted *p* < 0.001; all *q* < 0.012). These differences were expected because anemia status was defined using the sex-specific hemoglobin threshold and should not be interpreted as an independent analytical finding.

Serum iron was nominally lower in women with anemia than in those without anemia in the HFpEF group (6.4 versus 14.1 μmol/L; unadjusted *p* = 0.028; *q* = 0.168) and the HFrEF group (6.25 versus 12.55 μmol/L; unadjusted *p* = 0.020; *q* = 0.144). No corresponding difference was observed in HFmrEF (11.2 versus 13.3 μmol/L; unadjusted *p* = 0.383). Thus, the serum iron comparisons did not remain statistically significant after correction for multiple testing.

In women with HFpEF, plasma hypoxanthine was lower among patients with anemia than among those without anemia (153.5 versus 207.0; unadjusted *p* = 0.006); however, the association did not meet the false-discovery-rate threshold (*q* = 0.054). No differences in plasma hypoxanthine were observed according to anemia status in HFmrEF or HFrEF. Ferritin, platelet count, the remaining purine metabolites, and oxidative stress biomarkers did not differ significantly between women with and without anemia in any LVEF category after multiplicity correction. Although median ferritin was consistently lower in women with anemia, particularly in the HFpEF group (20.35 versus 58.35 μg/L), these descriptive findings cannot distinguish absolute from functional iron deficiency because transferrin saturation, transferrin or total iron-binding capacity, soluble transferrin receptor, and hepcidin were not measured.

Among men, median hemoglobin concentration was lower in patients with anemia than in those without anemia in HFpEF (107.0 versus 144.5 g/L), HFmrEF (117 versus 152 g/L), and HFrEF (116 versus 144 g/L; all unadjusted *p* < 0.001; all *q* < 0.009). As in women, these differences primarily reflect the hemoglobin-based definition of anemia. Platelet count was higher in men with anemia than in those without anemia in the HFpEF group (287 versus 203 × 10^9^/L; unadjusted *p* < 0.001; *q* < 0.009). No corresponding differences were found in HFmrEF or HFrEF. Serum iron was lower in men with anemia across all three LVEF categories. The median values in patients with and without anemia were 8.8 versus 18.1 μmol/L in HFpEF (unadjusted *p* = 0.004; *q* = 0.029), 7.2 versus 17.6 μmol/L in HFmrEF (unadjusted *p* = 0.015; *q* = 0.077), and 5.5 versus 12.0 μmol/L in HFrEF (unadjusted *p* = 0.006; *q* = 0.036). After false discovery rate correction, the associations remained statistically significant in the HFpEF and HFrEF groups but not in the HFmrEF group. Nevertheless, serum iron alone is insufficient to establish the presence or mechanism of iron deficiency. Ferritin, plasma purine metabolites, and oxidative stress biomarkers did not differ significantly between men with and without anemia in any LVEF category after correction for multiple testing. Overall, anemia was associated with lower serum iron in selected male subgroups, whereas evidence for anemia-related differences in purine metabolism or oxidative stress biomarkers was not statistically robust. These findings are associative and exploratory and do not establish distinct mechanisms of anemia in women and men.

### 3.4. Comparisons Between Women and Men Within LVEF Categories

Laboratory biomarkers were compared between women and men separately within each LVEF category (Table 4). The Benjamini–Hochberg procedure was applied across all 36 comparisons. Because these were stratified subgroup comparisons rather than formal interaction analyses, differences observed within an individual LVEF category were not interpreted as evidence of a sex-by-LVEF interaction.

Hemoglobin concentration was nominally higher in men than in women with HFpEF (132 versus 111 g/L; unadjusted *p* = 0.007), although this difference did not remain statistically significant after correction for multiple comparisons (*q* = 0.063). In the HFmrEF group, hemoglobin was higher in men than in women (148 versus 121 g/L; unadjusted *p* < 0.001; *q* < 0.036), and this difference remained statistically significant after FDR correction. No significant difference was observed in HFrEF (141 versus 132 g/L; unadjusted *p* = 0.154; *q* = 0.379). Serum iron was nominally higher in men than in women with HFpEF (13.30 versus 8.45 μmol/L; unadjusted *p* = 0.027), but the association did not remain significant after multiplicity correction (*q* = 0.140). Serum iron did not differ significantly between women and men in the HFmrEF or HFrEF groups. Ferritin concentrations also showed no significant sex-related differences in any LVEF category. Accordingly, these measurements do not support classification of the observed patterns as functional or absolute iron deficiency.

Several purine metabolism biomarkers showed nominally higher median concentrations in men. Plasma purines were nominally higher in men with HFmrEF (207 versus 165; unadjusted *p* = 0.032; *q* = 0.140) and HFrEF (212 versus 159; unadjusted *p* = 0.035; *q* = 0.140). Plasma adenine was nominally higher in men with HFmrEF (143.5 versus 105.5; unadjusted *p* = 0.012; *q* = 0.086). Plasma xanthine was nominally higher in men with HFmrEF (207.5 versus 157.5; unadjusted *p* = 0.005; *q* = 0.060) and HFrEF (222.0 versus 154.5; unadjusted *p* = 0.031; *q* = 0.140). Plasma uric acid was nominally higher in men with HFrEF (244 versus 173; unadjusted *p* = 0.003), but this comparison did not meet the FDR-adjusted significance threshold (*q* = 0.054).

Platelet count, plasma hypoxanthine, and oxidative stress biomarkers did not differ significantly between women and men in any LVEF category after correction for multiple comparisons. Overall, only the difference in hemoglobin concentration within the HFmrEF group remained statistically significant after FDR correction. The remaining biomarker differences should be regarded as exploratory and require confirmation in adequately powered analyses incorporating formal interaction terms and adjustment for relevant clinical confounders.

### 3.5. Exploratory Analysis of Purine Metabolism Biomarkers

An exploratory analysis compared plasma purine metabolism biomarkers between women and men with anemia within each LVEF category (Table 5). Only plasma concentrations are reported because the available results did not provide independently interpretable erythrocyte measurements with separate units and analytical characteristics. The previously used combined designation “erythrocyte/plasma” was therefore removed to avoid treating differently scaled values as independent measurements. Among patients with HFpEF, plasma purines, hypoxanthine, and xanthine did not differ between men and women (all unadjusted *p* ≥ 0.369). No statistically significant differences were observed in the HFmrEF group (all unadjusted *p* ≥ 0.095).

Among patients with HFrEF, median plasma concentrations were numerically higher in men than in women for total purines (213 versus 153; unadjusted *p* = 0.049), hypoxanthine (204 versus 124; unadjusted *p* = 0.049), and xanthine (231 versus 134; unadjusted *p* = 0.041). However, none of these comparisons remained statistically significant after Benjamini–Hochberg correction across the nine purine metabolism comparisons (all *q* = 0.147). These findings do not demonstrate that sex-related divergence in purine metabolism occurs specifically in HFrEF. The observed differences are hypothesis-generating and should be interpreted cautiously because of the small HFrEF subgroups (12 men and 8 women), the cross-sectional design, and the absence of formal sex-by-anemia-by-LVEF interaction testing.

### 3.6. Summary After Multiple-Testing Correction

After application of the Benjamini–Hochberg false discovery rate correction, the number of statistically significant findings was substantially reduced. None of the comparisons of laboratory biomarkers across LVEF categories remained statistically significant when women and men were analyzed separately (Table 2). In comparisons according to anemia status (Table 3), hemoglobin remained lower in patients with anemia across all sex and LVEF subgroups. However, this finding was expected because anemia was defined using sex-specific hemoglobin thresholds and therefore does not represent an independent association. Among men, a higher platelet count in patients with anemia and HFpEF remained statistically significant (*q* < 0.009). Lower serum iron in men with anemia also remained significant in the HFpEF (*q* = 0.029) and HFrEF (*q* = 0.036) groups, but not in HFmrEF (*q* = 0.077). No iron-related, purine metabolism, or oxidative stress biomarker other than hemoglobin remained significant according to anemia status among women.

In direct comparisons between women and men within the LVEF categories (Table 4), only the higher hemoglobin concentration in men with HFmrEF remained statistically significant (*q* < 0.036). The nominal differences in serum iron, purines, adenine, xanthine, and uric acid did not meet the FDR-adjusted significance threshold. Among patients with anemia, none of the sex-related differences in plasma purines, hypoxanthine, or xanthine remained statistically significant after correction across the nine exploratory comparisons (all *q* ≥ 0.147; Table 5). Consequently, the present results do not provide statistically robust evidence that sex-related divergence in purine metabolism occurs specifically in HFrEF. Overall, the multiplicity-adjusted findings support a limited number of descriptive associations, principally involving hemoglobin, serum iron, and platelet count. They do not establish distinct sex-specific mechanisms of iron deficiency, inflammation, oxidative stress, or purine metabolism. Furthermore, because the analyses were based on small stratified subgroups and did not include formal interaction testing or multivariable adjustment, the findings should be regarded as exploratory and hypothesis-generating.

## 4. Discussion

### 4.1. Principal Findings

This cross-sectional study explored hematological, iron-related, oxidative stress, and purine metabolism biomarkers according to sex, anemia status, and left ventricular ejection fraction (LVEF) category in patients with chronic heart failure (CHF). After correction for multiple testing, substantially fewer associations remained statistically significant than in the unadjusted analyses. First, none of the biomarker comparisons across heart failure with preserved ejection fraction (HFpEF), mildly reduced ejection fraction (HFmrEF), and reduced ejection fraction (HFrEF) remained statistically significant when women and men were analyzed separately. Second, hemoglobin concentrations were lower in patients classified as anemic across all sex and LVEF subgroups. However, this result was expected because anemia was defined using sex-specific hemoglobin thresholds and should not be interpreted as an independent finding. Among men, anemia remained associated with a higher platelet count in HFpEF and lower serum iron concentrations in HFpEF and HFrEF after false-discovery-rate (FDR) correction. Third, in direct comparisons between women and men, only the higher hemoglobin concentration among men with HFmrEF remained statistically significant after adjustment for multiple testing. Finally, none of the exploratory sex-related differences in plasma purines, hypoxanthine, or xanthine among patients with anemia remained statistically significant after FDR correction. Accordingly, the adjusted findings do not confirm the initially proposed distinction between inflammation-associated functional iron deficiency in women and absolute iron deficiency in men. They also do not demonstrate that sex-related divergence in purine metabolism occurs specifically in HFrEF. The observed patterns should instead be interpreted as exploratory differences in the measured biomarkers that require confirmation in adequately powered studies using formal interaction testing and multivariable analyses.

### 4.2. Interpretation of Iron-Related Biomarkers

Anemia and iron deficiency are related but distinct conditions in CHF. Anemia may result from renal dysfunction, impaired erythropoietin production or responsiveness, inflammation-associated iron restriction, nutritional deficiencies, blood loss, hemodilution, bone marrow disorders, or treatment-related factors. Conversely, iron deficiency may be present without anemia. Therefore, hemoglobin concentration cannot be used as a surrogate marker of iron status.

The present study measured serum iron and ferritin but did not include transferrin saturation (TSAT), transferrin, total iron-binding capacity, soluble transferrin receptor, or hepcidin. This substantially limits mechanistic interpretation. Serum iron is affected by fasting status, diurnal variation, recent dietary intake, inflammation, and acute illness. Ferritin reflects body iron stores but is also an acute-phase reactant and may increase in the presence of inflammation, hepatic dysfunction, infection, or other systemic disorders. Importantly, the median ferritin concentration among women with HFpEF was 33.0 μg/L and decreased to 20.35 μg/L among women with HFpEF and anemia. These comparatively low values are difficult to reconcile with a conclusion that iron stores were preserved in the presence of inflammatory iron sequestration. Instead, they may be compatible with reduced or depleted iron stores, although the absence of TSAT and other iron indices prevents definitive classification. Contemporary evidence has questioned reliance on ferritin alone and increasingly emphasizes TSAT as a clinically informative marker of insufficient circulating iron availability in heart failure [18]. The lower serum iron concentrations identified in men with anemia and HFpEF or HFrEF remained statistically significant after FDR correction. Nevertheless, these associations do not establish absolute iron deficiency. Without TSAT, transferrin-related measurements, and preferably soluble transferrin receptor and hepcidin, it is not possible to determine whether the observed reductions reflect depletion of iron stores, inflammation-associated iron restriction, renal dysfunction, blood loss, nutritional factors, or a combination of these mechanisms.

Recent research has also highlighted a broader network of iron-homeostasis regulators beyond ferritin and hepcidin. Biomarkers such as matriptase-2 and neogenin-1 may provide additional information regarding regulation of the hepcidin–ferroportin pathway [19]. However, the cited evidence was obtained in pediatric patients with leukemia and cannot be directly extrapolated to adults with CHF. Its relevance to heart failure should therefore be regarded as a rationale for future investigation rather than an explanation of the present findings.

### 4.3. Sex, Heart Failure Phenotype, and Inflammation

Women and men with CHF frequently differ in age, body composition, LVEF distribution, disease etiology, renal function, comorbidity burden, hormonal status, and treatment. Women are more frequently represented in HFpEF cohorts, whereas ischemic cardiomyopathy and HFrEF are generally more common among men [5,6]. These characteristics may influence hemoglobin, ferritin, serum iron, platelet count, purine metabolites, and oxidative stress markers independently of biological sex. The present findings should therefore not be interpreted as demonstrating that inflammation is predominantly a female mechanism of CHF-associated anemia. Inflammation contributes to the pathophysiology of CHF in both sexes. Moreover, the available evidence does not demonstrate a uniform female predominance in inflammatory activity. In the PROMIS-HFpEF cohort, Simmonds et.al. described sex-related differences in the proteomic correlates of coronary microvascular dysfunction, with inflammatory chemokine and cytokine pathways being more prominent among men and remodeling- and fibrosis-related pathways being more prominent among women [20]. Other research has suggested that increased inflammatory activity may have a stronger adverse prognostic association in men with heart failure [21]. Experimental and clinical evidence indicates that sex hormones may modify innate and adaptive immune responses. Estrogens, androgens, menopausal status, age-related hormonal changes, and the underlying disease etiology may influence inflammation and fibrosis, but these relationships are complex and context-dependent [22]. Tumor necrosis factor-alpha overexpression and other inflammatory processes have also been associated with worse New York Heart Association (NYHA) functional class, higher natriuretic peptide concentrations, and poorer quality of life in both women and men [23]. Consequently, inflammation cannot be assigned exclusively to either sex.

Age-related clonal hematopoiesis represents another emerging potential link between hematological abnormalities, inflammation, and cardiovascular disease. A recent systematic review and meta-analysis reported associations between clonal hematopoiesis, incident heart failure, and adverse outcomes among patients with established heart failure [24]. This evidence raises the possibility that age-associated hematopoietic alterations may contribute to inflammatory heterogeneity in CHF. However, clonal hematopoiesis was not evaluated in the present study. Obesity and metabolic disease may further modify inflammatory activity, particularly in HFpEF. Contemporary research emphasizes not only the activation of inflammatory pathways but also the failure of inflammation-resolution mechanisms in obesity-related cardiovascular disease [25]. Because body mass index, diabetes, inflammatory biomarkers, and related metabolic variables were not incorporated into adjusted models in the present analysis, inflammation cannot be invoked as the demonstrated cause of the observed sex-related biomarker patterns.

### 4.4. Purine Metabolism and Oxidative Stress Biomarkers

Purine metabolites have biological plausibility as markers of altered cellular energy turnover, oxidative stress, ischemia, or reduced oxygen availability. Hypoxanthine and xanthine are generated during adenine nucleotide degradation, whereas xanthine oxidoreductase activity may contribute to uric acid production and reactive oxygen species generation [3,13]. Anemia and impaired cardiac output could theoretically affect these pathways through reduced tissue oxygen delivery. In the present study, unadjusted analyses showed numerically higher plasma concentrations of total purines, hypoxanthine, and xanthine in anemic men than in anemic women with HFrEF. However, these comparisons were based on only 12 men and 8 women, and the corresponding unadjusted *p*-values ranged from 0.041 to 0.049. After FDR correction, all three comparisons had a *q*-value of 0.147 and were no longer statistically significant. Therefore, these findings are particularly vulnerable to random variation and cannot support the assertion that sex-related metabolic divergence emerges specifically in HFrEF. The absence of statistically significant differences in HFpEF and HFmrEF cannot be interpreted as evidence that women and men are metabolically equivalent within these categories. The small subgroup sizes provide limited ability to distinguish the absence of an association from insufficient statistical power. Furthermore, separate significance tests within subgroups do not demonstrate that an association differs between the subgroups. Such a conclusion would require formal sex-by-anemia-by-LVEF interaction testing.

Purine concentrations may also be influenced by renal clearance, ischemic etiology, dietary intake, alcohol consumption, medication use, tissue mass, sampling conditions, and the clinical stability of heart failure. These factors were not comprehensively controlled in the present analysis. Similarly, the absence of adjusted differences in oxidative modification of proteins (OMB) and advanced oxidation protein products (AOPPs) does not exclude oxidative stress as a component of CHF pathophysiology. It indicates only that the biomarkers measured in this study did not demonstrate robust differences between the analyzed groups.

### 4.5. Relationship to Contemporary Evidence on Iron Deficiency Treatment

The clinical implications of iron deficiency in heart failure remain an evolving area. The 2023 focused update of the European Society of Cardiology guidelines supports the assessment of iron status and the considered use of intravenous iron in selected symptomatic patients with HFrEF or HFmrEF and iron deficiency, particularly to improve symptoms, exercise capacity, and quality of life and potentially reduce the risk of heart failure hospitalization [26]. These recommendations are based on definitions incorporating ferritin and TSAT rather than serum iron alone. In the HEART-FID trial, intravenous ferric carboxymaltose was evaluated in a large population of patients with HFrEF and iron deficiency but did not meet the prespecified statistical threshold for its primary hierarchical outcome [27]. The FAIR-HFpEF trial was terminated early after the enrollment of only 39 participants. Although ferric carboxymaltose improved six-minute walking distance, the trial was insufficiently powered to establish its effects on symptoms, quality of life, or clinical events in the broader HFpEF population [28]. The FAIR-HF2 trial subsequently did not demonstrate statistically significant reductions in its prespecified primary clinical outcomes with ferric carboxymaltose in patients with HFrEF and iron deficiency [29].

Together, these studies indicate that the therapeutic consequences of iron deficiency vary according to the outcome examined and remain incompletely defined across the LVEF spectrum. The present observational study did not evaluate intravenous iron therapy, treatment response, hospitalization, mortality, exercise capacity, or quality of life. It therefore cannot support sex-specific diagnostic thresholds or treatment recommendations. Its findings should not alter current guideline-based assessment, which requires a more complete evaluation of iron status.

### 4.6. Strengths and Limitations

A strength of this study is the joint exploratory assessment of hematological, iron-related, oxidative stress, and purine metabolism biomarkers according to sex, anemia status, and LVEF category within a single CHF cohort. The use of sex-specific World Health Organization thresholds for anemia and the reporting of multiplicity-adjusted *q*-values also improve the interpretability of the findings.

Several important limitations must be acknowledged. First, the cross-sectional design precludes conclusions regarding progression, temporal sequence, compensation, or metabolic decompensation. Differences among HFpEF, HFmrEF, and HFrEF represent comparisons between different patients at a single assessment and not longitudinal changes within the same individuals. Second, the cohort was divided into as many as 12 subgroups, several of which contained only 8–18 participants. No a priori sample-size calculation was performed. Consequently, the effect estimates are imprecise, statistical power is limited, and both nominally significant and nonsignificant findings must be interpreted cautiously. Third, separate subgroup comparisons do not formally establish sex-specific effects. This study did not perform sex-by-LVEF, sex-by-anemia, or sex-by-anemia-by-LVEF interaction analyses. Multivariable adjustment for age, renal function, body mass index, diabetes, hypertension, ischemic etiology, NYHA functional class, natriuretic peptides, and heart failure therapies was also not available. Residual and unmeasured confounding is therefore likely. Fourth, iron status was incompletely characterized. The absence of TSAT, transferrin or total iron-binding capacity, soluble transferrin receptor, hepcidin, and inflammatory biomarkers prevents differentiation between absolute iron deficiency and inflammation-associated iron restriction. Serum iron and ferritin alone are insufficient to establish these mechanisms. Fifth, alternative causes and modifiers of anemia were not comprehensively documented. These include recent oral or intravenous iron therapy, erythropoiesis-stimulating agents, blood transfusion, vitamin B12 or folate deficiency, occult or previous bleeding, advanced chronic kidney disease, autoimmune disease, previous anticancer chemotherapy, and menopausal status. Treatment with renin–angiotensin–aldosterone system inhibitors and other CHF therapies may also affect hematological or renal variables. Sixth, the requirement for the availability of a core laboratory panel may have introduced selection bias. Missing biomarker values were handled using available-case analysis, which may have further affected group comparability if the data were not missing completely at random. Limited information regarding the recruitment setting, clinical stability, and outpatient versus hospitalized or decompensated status also restricts the generalizability of the findings.

Finally, the analytical methodology and measurement units for purine metabolites, OMB, and AOPPs require complete reporting. Until independently measured plasma and erythrocyte values, calibration procedures, intra- and inter-assay variability, and quality-control characteristics are fully documented, compartment-specific interpretation should be approached cautiously.

### 4.7. Implications and Future Research

Future studies should prospectively recruit adequately powered cohorts and prespecify primary and exploratory outcomes. Sex-by-LVEF and sex-by-anemia interaction terms should be evaluated, together with a three-way sex-by-anemia-by-LVEF interaction for purine metabolism outcomes. Where appropriate, LVEF should also be modeled as a continuous variable, potentially using restricted cubic splines, to reduce the information loss caused by clinical categorization.

Adjusted analyses should incorporate age, kidney function, body mass index, diabetes, hypertension, heart failure etiology, NYHA functional class, natriuretic peptides, clinical setting, and relevant therapies. Age-stratified analyses and documentation of menopausal status would help distinguish potential hormonal effects from age- and comorbidity-related differences. A more complete iron panel should include ferritin, TSAT, transferrin or total iron-binding capacity, soluble transferrin receptor, hepcidin, and inflammatory markers such as C-reactive protein and interleukin-6. Investigation of emerging regulators, including matriptase-2, neogenin-1, and other components of the hepcidin–ferroportin pathway, may provide additional mechanistic information, although their relevance to CHF must first be established [19].

Purine metabolism analyses require standardized sampling, clearly reported analytical units, separate reporting of plasma and erythrocyte measurements, assay validation, and adjustment for renal function and ischemic etiology. Longitudinal studies are needed to determine whether changes in purine metabolites precede or follow changes in cardiac function, anemia, clinical congestion, or treatment. Until such evidence becomes available, the present findings should remain hypothesis-generating and should not be used to justify sex-specific clinical management.

## 5. Conclusions

In this cross-sectional cohort of patients with chronic heart failure, hematological, iron-related, oxidative stress, and purine metabolism biomarkers varied according to sex, anemia status, and LVEF category in the unadjusted subgroup analyses. However, most differences did not remain statistically significant after correction for multiple testing.

Among men, anemia was associated with lower serum iron concentrations in the HFpEF and HFrEF groups and with a higher platelet count in the HFpEF group after false discovery rate correction. In direct comparisons between sexes, only the higher hemoglobin concentration in men with HFmrEF remained statistically significant after adjustment for multiple testing. The initially observed differences in plasma purines, hypoxanthine, and xanthine between anemic women and men with HFrEF did not remain statistically significant after multiplicity correction.

Because TSAT, transferrin-related indices, soluble transferrin receptor, hepcidin, and inflammatory biomarkers were not measured, the present findings do not permit differentiation between absolute iron deficiency and inflammation-associated functional iron restriction. They also do not establish sex-specific mechanisms of anemia or purine metabolism alterations in CHF.

The findings should therefore be regarded as exploratory and hypothesis-generating. Prospective studies with larger samples, comprehensive clinical characterization, complete assessment of iron and inflammatory status, and formal sex-by-anemia and sex-by-LVEF interaction analyses are required to determine whether clinically meaningful sex-related differences exist. The present results do not provide sufficient evidence to support sex-specific diagnostic thresholds or treatment strategies for anemia and iron deficiency in patients with CHF.

## Figures and Tables

**Table 1:** Distribution of Patients According to Sex, Anemia Status, and LVEF Category.

LVEF Category	Women Without Anemia, *n*	Women with Anemia, *n*	Women, Total *n*	Men Without Anemia, *n*	Men with Anemia, *n*	Men, Total *n*	Overall, *n*
**HFpEF**	8	18	26	16	11	27	53
**HFmrEF**	13	13	26	29	9	38	64
**HFrEF**	14	8	22	27	13	40	62
**Total**	35	39	74	72	33	105	179

**Table 2:** Laboratory Biomarkers Across LVEF Categories Stratified by Sex.

A. Women					
Biomarker	HFpEF, *n*; median	HFmrEF, *n*; median	HFrEF, *n*; median	Raw *p*	BH-adjusted *q*
Platelets, ×10^9^/L	26; 276.5	26; 217.0	22; 202.5	0.006	0.084
Leukocytes, ×10^9^/L	26; 6.25	26; 6.45	22; 7.15	0.781	0.911
Hemoglobin, g/L	26; 111	26; 121	22; 132	0.035	0.168
Erythrocytes, ×10^12^/L	26; 4.200	26; 4.345	22; 4.545	0.279	0.646
Ferritin, μg/L	26; 33.00	26; 90.50	22; 127.15	0.036	0.168
Serum iron, μmol/L	26; 8.45	26; 12.85	22; 10.80	0.071	0.248
Purines, plasma	26; 188.5	26; 165.0	22; 159.0	0.424	0.660
Hypoxanthine, plasma	26; 159.5	26; 133.0	22; 137.5	0.573	0.802
Adenine, plasma	26; 138.5	26; 105.5	22; 120.5	0.323	0.646
Xanthine, plasma	26; 186.5	26; 157.5	22; 154.5	0.190	0.532
Uric acid, plasma	26; 208.5	26; 184.0	22; 173.0	0.402	0.660
OMB, erythrocytes	26; 0.1545	26; 0.1590	22; 0.1565	0.970	0.970
OMB, plasma	26; 1.33766	26; 1.37662	22; 1.35498	0.970	0.970
AOPPs	26; 0.2030	26; 0.2010	22; 0.2025	0.654	0.832
B. Men					
Platelets, ×10^9^/L	27; 230.0	38; 227.0	40; 206.5	0.115	0.460
Hemoglobin, g/L	27; 132	38; 148	40; 141	0.021	0.132
Ferritin, μg/L	26; 98.80	38; 113.15	40; 125.00	0.450	0.771
Serum iron, μmol/L	26; 13.30	38; 15.30	40; 9.50	0.022	0.132
Purines, plasma	26; 229.0	38; 207.0	39; 212.0	0.879	0.879
Hypoxanthine, plasma	26; 176.0	38; 167.5	39; 173.0	0.660	0.802
Adenine, plasma	26; 141.0	38; 143.5	39; 142.0	0.735	0.802
Xanthine, plasma	26; 195.0	38; 207.5	39; 222.0	0.348	0.771
Uric acid, plasma	26; 246.0	38; 216.5	39; 244.0	0.444	0.771
OMB, erythrocytes	26; 0.178	38; 0.154	39; 0.178	0.692	0.802
OMB, plasma	26; 1.54113	38; 1.33333	39; 1.54113	0.692	0.802
AOPPs	26; 0.2120	38; 0.1995	39; 0.1680	0.168	0.504

**Table 3:** Comparison of Laboratory Biomarkers Between Patients With and Without Anemia Stratified by Sex and LVEF Category.

Sex	Biomarker	LVEF Category	No Anemia, *n*	No Anemia, Median	Anemia, *n*	Anemia, Median	Raw *p*	Adjusted *q*
Women	Ferritin, μg/L	HFpEF	8	58.35	18	20.35	0.113	0.452
Women	Ferritin, μg/L	HFmrEF	13	108.80	13	70.70	0.682	0.877
Women	Ferritin, μg/L	HFrEF	14	127.15	8	99.65	0.865	0.972
Women	Platelets, ×10^9^/L	HFpEF	8	274.5	18	276.5	0.846	0.972
Women	Platelets, ×10^9^/L	HFmrEF	13	212.0	13	232.0	0.918	0.972
Women	Platelets, ×10^9^/L	HFrEF	14	208.0	8	194.5	0.375	0.649
Women	Hemoglobin, g/L	HFpEF	8	131.0	18	102.0	<0.001	<0.012
Women	Hemoglobin, g/L	HFmrEF	13	136.0	13	103.0	<0.001	<0.012
Women	Hemoglobin, g/L	HFrEF	14	141.5	8	103.5	<0.001	<0.012
Women	Serum iron, μmol/L	HFpEF	8	14.10	18	6.40	0.028	0.168
Women	Serum iron, μmol/L	HFmrEF	13	13.30	13	11.20	0.383	0.649
Women	Serum iron, μmol/L	HFrEF	14	12.55	8	6.25	0.020	0.144
Women	Purines, plasma	HFpEF	8	207.0	18	182.0	0.211	0.557
Women	Purines, plasma	HFmrEF	13	163.0	13	166.0	0.397	0.649
Women	Purines, plasma	HFrEF	14	159.0	8	153.5	0.474	0.683
Women	Hypoxanthine, plasma	HFpEF	8	207.0	18	153.5	0.006	0.054
Women	Hypoxanthine, plasma	HFmrEF	13	134.0	13	132.0	0.663	0.877
Women	Hypoxanthine, plasma	HFrEF	14	142.5	8	124.5	0.433	0.649
Women	Adenine, plasma	HFpEF	8	138.5	18	138.0	0.617	0.854
Women	Adenine, plasma	HFmrEF	13	106.0	13	102.0	0.798	0.958
Women	Adenine, plasma	HFrEF	14	132.0	8	93.0	0.433	0.649
Women	Xanthine, plasma	HFpEF	8	199.0	18	179.5	0.232	0.557
Women	Xanthine, plasma	HFmrEF	13	156.0	13	159.0	0.412	0.649
Women	Xanthine, plasma	HFrEF	14	171.0	8	134.0	0.322	0.644
Women	Uric acid, plasma	HFpEF	8	232.5	18	187.0	0.255	0.574
Women	Uric acid, plasma	HFmrEF	13	175.0	13	194.0	0.137	0.493
Women	Uric acid, plasma	HFrEF	14	155.0	8	189.5	0.322	0.644
Women	OMB, erythrocytes	HFpEF	8	0.1190	18	0.1565	0.055	0.247
Women	OMB, erythrocytes	HFmrEF	13	0.1690	13	0.1440	0.980	0.980
Women	OMB, erythrocytes	HFrEF	14	0.1910	8	0.1445	0.219	0.557
Women	OMB, plasma	HFpEF	8	1.03030	18	1.35498	0.055	0.247
Women	OMB, plasma	HFmrEF	13	1.46320	13	1.24675	0.980	0.980
Women	OMB, plasma	HFrEF	14	1.65368	8	1.25108	0.219	0.557
Women	AOPPs	HFpEF	8	0.2245	18	0.1985	0.912	0.972
Women	AOPPs	HFmrEF	13	0.2050	13	0.1970	0.758	0.941
Women	AOPPs	HFrEF	14	0.2285	8	0.1610	0.162	0.530
Men	Ferritin, μg/L	HFpEF	15	99.5	11	70.9	0.568	0.757
Men	Ferritin, μg/L	HFmrEF	29	116.2	9	80.1	0.514	0.757
Men	Ferritin, μg/L	HFrEF	27	131.0	13	84.3	0.862	0.970
Men	Platelets, ×10^9^/L	HFpEF	16	203.0	11	287.0	<0.001	<0.009
Men	Platelets, ×10^9^/L	HFmrEF	29	224.0	9	244.0	0.945	1.000
Men	Platelets, ×10^9^/L	HFrEF	27	200.0	13	220.0	0.908	0.991
Men	Hemoglobin, g/L	HFpEF	16	144.5	11	107.0	<0.001	<0.009
Men	Hemoglobin, g/L	HFmrEF	29	152.0	9	117.0	<0.001	<0.009
Men	Hemoglobin, g/L	HFrEF	27	144.0	13	116.0	<0.001	<0.009
Men	Serum iron, μmol/L	HFpEF	15	18.1	11	8.8	0.004	0.029
Men	Serum iron, μmol/L	HFmrEF	29	17.6	9	7.2	0.015	0.077
Men	Serum iron, μmol/L	HFrEF	27	12.0	13	5.5	0.006	0.036
Men	Purines, plasma	HFpEF	15	232.0	11	184.0	0.254	0.703
Men	Purines, plasma	HFmrEF	29	205.0	9	209.0	0.525	0.757
Men	Purines, plasma	HFrEF	27	211.0	12	213.0	0.563	0.757
Men	Hypoxanthine, plasma	HFpEF	15	186.0	11	154.0	0.568	0.757
Men	Hypoxanthine, plasma	HFmrEF	29	171.0	9	162.0	0.718	0.879
Men	Hypoxanthine, plasma	HFrEF	27	170.0	12	204.0	0.338	0.757
Men	Adenine, plasma	HFpEF	15	151.0	11	131.0	0.378	0.757
Men	Adenine, plasma	HFmrEF	29	137.0	9	150.0	0.757	0.879
Men	Adenine, plasma	HFrEF	27	142.0	12	165.0	0.361	0.757
Men	Xanthine, plasma	HFpEF	15	196.0	11	184.0	0.324	0.757
Men	Xanthine, plasma	HFmrEF	29	206.0	9	221.0	0.559	0.757
Men	Xanthine, plasma	HFrEF	27	209.0	12	231.5	0.411	0.757
Men	Uric acid, plasma	HFpEF	15	281.0	11	236.0	0.254	0.703
Men	Uric acid, plasma	HFmrEF	29	215.0	9	230.0	0.514	0.757
Men	Uric acid, plasma	HFrEF	27	221.0	12	278.5	0.110	0.440
Men	OMB, erythrocytes	HFpEF	15	0.165	11	0.202	0.755	0.879
Men	OMB, erythrocytes	HFmrEF	29	0.147	9	0.164	0.430	0.757
Men	OMB, erythrocytes	HFrEF	27	0.161	12	0.231	0.186	0.609
Men	OMB, plasma	HFpEF	15	1.42857	11	1.74892	0.755	0.879
Men	OMB, plasma	HFmrEF	29	1.27273	9	1.41991	0.430	0.757
Men	OMB, plasma	HFrEF	27	1.39394	12	2.00000	0.186	0.609
Men	AOPPs	HFpEF	15	0.2270	11	0.1970	1.000	1.000
Men	AOPPs	HFmrEF	29	0.2100	9	0.1640	1.000	1.000
Men	AOPPs	HFrEF	27	0.2060	12	0.1515	0.100	0.440

**Table 4:** Comparison of Laboratory Biomarkers Between Women and Men Within LVEF Categories.

Biomarker	LVEF Category	Men, *n*	Men, Median	Women, *n*	Women, Median	Raw *p*	Adjusted *q*
Ferritin, μg/L	HFpEF	26	98.80	26	33.00	0.081	0.224
	HFmrEF	38	113.15	26	90.50	0.707	0.848
	HFrEF	40	125.00	22	127.15	0.627	0.806
Platelets, ×10^9^/L	HFpEF	27	230.0	26	276.5	0.200	0.450
	HFmrEF	38	227.0	26	217.0	0.440	0.720
	HFrEF	40	206.5	22	202.5	0.889	0.939
Hemoglobin, g/L	HFpEF	27	132.0	26	111.0	0.007	0.063
	HFmrEF	38	148.0	26	121.0	<0.001	<0.036
	HFrEF	40	141.0	22	132.0	0.154	0.379
Serum iron, μmol/L	HFpEF	26	13.30	26	8.45	0.027	0.140
	HFmrEF	38	15.30	26	12.85	0.440	0.720
	HFrEF	40	9.50	22	10.80	0.785	0.862
Purines, plasma	HFpEF	26	229.0	26	188.5	0.268	0.568
	HFmrEF	38	207.0	26	165.0	0.032	0.140
	HFrEF	39	212.0	22	159.0	0.035	0.140
Hypoxanthine, plasma	HFpEF	26	176.0	26	159.5	0.314	0.628
	HFmrEF	38	167.5	26	133.0	0.052	0.170
	HFrEF	39	173.0	22	159.0	0.413	0.720
Adenine, plasma	HFpEF	26	141.0	26	138.5	0.687	0.848
	HFmrEF	38	143.5	26	105.5	0.012	0.086
	HFrEF	39	142.0	22	120.5	0.158	0.379
Xanthine, plasma	HFpEF	26	195.0	26	186.5	0.602	0.803
	HFmrEF	38	207.5	26	157.5	0.005	0.060
	HFrEF	39	222.0	22	154.5	0.031	0.140
Uric acid, plasma	HFpEF	26	246.0	26	208.5	0.050	0.170
	HFmrEF	38	216.5	26	184.0	0.076	0.224
	HFrEF	39	244.0	22	173.0	0.003	0.054
OMB, erythrocytes	HFpEF	26	0.1780	26	0.1545	0.577	0.799
	HFmrEF	38	0.1540	26	0.1590	0.790	0.862
	HFrEF	39	0.1780	22	0.1565	0.485	0.728
OMB, plasma	HFpEF	26	1.54113	26	1.33766	0.577	0.799
	HFmrEF	38	1.33333	26	1.37662	0.790	0.862
	HFrEF	39	1.54113	22	1.35498	0.485	0.728
AOPPs	HFpEF	26	0.2120	26	0.2030	0.913	0.939
	HFmrEF	38	0.1995	26	0.2010	0.962	0.962
	HFrEF	39	0.1680	22	0.2025	0.333	0.631

Data are presented as the available-case sample size and median. Women and men were compared within each LVEF category using the Mann–Whitney U test. Raw *p*-values were adjusted across all 36 comparisons using the Benjamini–Hochberg false-discovery-rate procedure. The exact raw *p*-value reported as *p* < 0.001 was unavailable; therefore, its adjusted value is presented as a conservative upper bound of *q* < 0.036. A *q*-value < 0.05 was considered statistically significant. These stratified comparisons do not constitute formal evidence of a sex-by-LVEF interaction.

**Table 5:** Exploratory Comparison of Plasma Purine metabolism Biomarkers Between Women and Men With Anemia.

Biomarker	LVEF Category	Men, *n*	Men, Median	Women, *n*	Women, Median	Raw *p*	Adjusted *q*
Purines, plasma	HFpEF	11	184	18	182	0.608	0.684
	HFmrEF	9	209	13	166	0.217	0.391
	HFrEF	12	213	8	153	0.049	0.147
Hypoxanthine, plasma	HFpEF	11	154	18	153	0.369	0.474
	HFmrEF	9	162	13	132	0.301	0.452
	HFrEF	12	204	8	124	0.049	0.147
Xanthine, plasma	HFpEF	11	184	18	179	0.964	0.964
	HFmrEF	9	221	13	159	0.095	0.214
	HFrEF	12	231	8	134	0.041	0.147

Data are presented as the available-case sample size and median. Women and men with anemia were compared within each LVEF category using the Mann–Whitney U test. Raw *p*-values were adjusted across the nine comparisons using the Benjamini–Hochberg false-discovery-rate procedure. A *q*-value < 0.05 was considered statistically significant. None of the purine metabolism comparisons remained statistically significant after FDR correction. The subgroup comparisons were exploratory and do not constitute a formal sex-by-anemia-by-LVEF interaction analysis.

## Data Availability

The data presented in this study are not publicly available due to ethical and privacy restrictions but are available from the corresponding author upon reasonable request.

## Abbreviations

The following abbreviations are used in this manuscript:

Abbreviation	Definition
CHF	Chronic heart failure
FDR	False discovery rate
HFmrEF	Heart failure with mildly reduced ejection fraction
HFpEF	Heart failure with preserved ejection fraction
HFrEF	Heart failure with reduced ejection fraction
LVEF	Left ventricular ejection fraction
